# Laxative effect of repeated Daiokanzoto is attributable to decrease in aquaporin-3 expression in the colon

**DOI:** 10.1007/s11418-018-1174-1

**Published:** 2018-01-27

**Authors:** Risako Kon, Miho Yamamura, Yukari Matsunaga, Hiroshi Kimura, Moe Minami, Saki Kato, Nobutomo Ikarashi, Kiyoshi Sugiyama

**Affiliations:** 1 Global Research Center for Innovative Life Science, Hoshi University, Tokyo, Japan; 2 Division of Applied Pharmaceutical Education and Research, Hoshi University, Tokyo, Japan; 3 Department of Clinical Pharmacokinetics, Hoshi University, 2-4-41 Ebara, Shinagawa-ku, Tokyo, 142-8501 Japan; 4 Department of Functional Molecular Kinetics, Hoshi University, 2-4-41 Ebara, Shinagawa-ku, Tokyo, 142-8501 Japan

**Keywords:** Daiokanzoto, Aquaporin, Colon, Bile acid, Microbiota

## Abstract

Daiokanzoto (DKT) exerts its laxative effect via colonic inflammation caused by sennoside A in Daio (rhubarb). Previously, we showed that the laxative effect of sennoside A is related to decreased aquaporin-3 (AQP3) expression in mucosal epithelial cells due to colonic inflammation. We also found that a combination of glycyrrhizin, an ingredient in Kanzo (glycyrrhiza), and sennoside A attenuates the inflammatory response induced by sennoside A and reduces its laxative effect. These findings indicate that DKT may be a long-term treatment for chronic constipation, but there is no evidence supporting this hypothesis. In this study, we analyzed the laxative effect of repeated DKT administration, focusing on AQP3 expression in the colon. After rats were treated for 7 days, decreased AQP3 expression and the onset of diarrhea were observed in the DKT group, but were not seen in the Daio group either. Although the relative abundance of gut microbiota after repeated DKT administration was similar to that after control treatment, Daio reduced *Lactobacillaceae*, *Bifidobacteriaceae*, and *Bacteroidaceae* levels and markedly increased *Lachnospiraceae* levels. In this study, we show that DKT has a sustained laxative effect, even upon repeated use, probably because it maintains decreased AQP3 expression and gut microbiota homeostasis. This outcome therefore indicates that DKT can be used as a long-term treatment for chronic constipation.

**Electronic supplementary material:**

The online version of this article (10.1007/s11418-018-1174-1) contains supplementary material, which is available to authorized users.

## Introduction

Daiokanzoto (DKT) is a Kampo medicine composed of two crude drugs, Daio (rhubarb) and Kanzo (glycyrrhiza), that can be repeatedly used for a long period to relieve constipation or related symptoms. The laxative effect of DKT is thought to be induced by sennoside A, the major ingredient of Daio. Ewe et al. [1] reported that when sennoside A was administered to mice, rheinanthrone was formed in the colon, and prostaglandin E_2_ production was increased, promoting peristaltic movements. Previously, we showed that the active metabolite of sennoside A, rheinanthrone, activates macrophages to exert its laxative effect through the reduced expression of aquaporin-3 (AQP3), a water channel in mucosal epithelial cells of the colon. We also found that pretreatment of rats with indomethacin, an anti-inflammatory drug, suppressed the sennoside A-induced decrease in AQP3 expression, which weakened the laxative effect of sennoside A [2].

Kanzo, another component of DKT, contains glycyrrhizin and has an anti-inflammatory effect. Thus, DKT is thought to have less severe adverse effects related to inflammation (e.g., painful defecation and abdominal pain) than senna or sennoside agent. We found that a combination of sennoside A and glycyrrhizin attenuated the inflammatory reaction in the colon and the laxative effect of sennoside A, indicating that glycyrrhizin controls the laxative effect (R. Kon, et al., Trad. Kampo Med., 2018). However, a previous report stated that Kanzo enhances the laxative effect of sennoside A [3], and the role of Kanzo in DKT is not clearly understood.

In general, the long-term use of stimulant laxatives such as Daio-containing drugs (senna), and the active ingredient sennoside causes drug resistance, whereby the therapeutic response is reduced. It has also been reported that the long-term use of these drugs causes adverse reactions, including cathartic colon and melanosis coli, which may progress to intestinal pseudo-obstruction or colon cancer [4–6]. Thus, stimulant laxatives such as Daio and senna are unsuitable for long-term use. DKT has a similar laxative effect as senna and sennoside but a lower incidence of adverse effects [7]. Based on these findings, we hypothesized that DKT, which contains the anti-inflammatory compound Kanzo, would have fewer adverse effects than senna or sennoside, which may enable its long-term use. However, we have not obtained scientific evidence for this hypothesis. In this study, we performed animal experiments to investigate whether DKT has efficacy under conditions of prolonged use and obtained scientific evidence of its usefulness as a therapeutic for chronic constipation. In addition, we clarified the roles of Kanzo in DKT. Briefly, Daio or DKT was administered orally to rats once (single dose) or for 7 days to compare the laxative effect using colon AQP3 expression as an index. We also analyzed the mechanism responsible for the change in AQP3 expression upon repeated administration of DKT.

## Materials and methods

### Materials

Daio extract, Kanzo extract, and DKT extract were purchased from Tsumura and Co. (Tokyo, Japan). Bovine serum albumin and TRI reagent were purchased from Sigma-Aldrich Corp. (St. Louis, MO, USA). Rabbit anti-rat AQP3 antibody was purchased from Alomone Labs (Jerusalem, Israel). Donkey anti-rabbit IgG-HRP and ECL Prime Western blotting detection reagents were purchased from GE Healthcare (Chalfont St. Giles, UK). All real-time PCR primers were purchased from Hokkaido System Science Co., Ltd. (Hokkaido, Japan). The High-Capacity cDNA Reverse Transcription kit was purchased from Applied Biosystems (Foster City, CA, USA). SsoAdvanced SYBR Green Supermix was purchased from Bio-Rad Laboratories (Hercules, CA, USA).

### Three-dimensional HPLC analysis

**Fig. 1:**
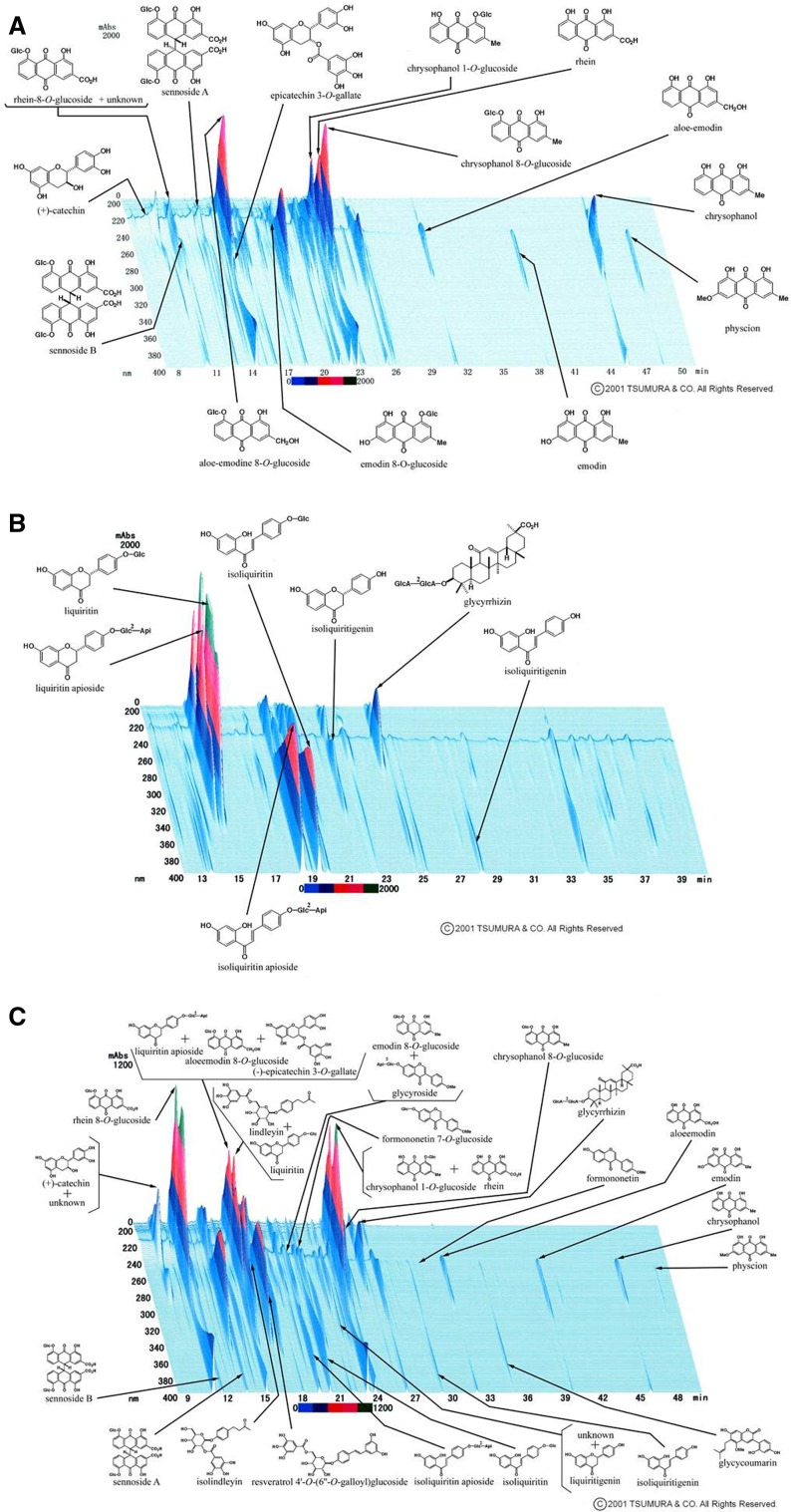
Three-dimensional HPLC chart of methanol solution, Daio (**a**), Kanzo (**b**), and DKT (**c**)

A granule of Daio (1.0 g), Kanzo (1.0 g), or DKT (1.0 g) was extracted with methanol under ultrasonication for 30 min and centrifuged at 3000 rpm for 5 min. The supernatant was filtered through a membrane and then submitted for HPLC analysis. The three-dimensional HPLC charts of the Daio, Kanzo, and DKT solutions are shown in Fig. 1.

### Animals

Male Wistar rats (8 weeks old) were purchased from Japan SLC, Inc. (Shizuoka, Japan). The rats were housed at 24 ± 1 °C and 55 ± 1% humidity with 12 h of light (08:00–20:00). The study was approved and conducted in accordance with the Hoshi University Guiding Principles for the Care and Use of Laboratory Animals.

### Treatments

Daio (1 g/kg), Kanzo (0.5 g/kg) or DKT (1.5 g/kg) was administered orally to rats once or for 7 days. At 5 h after the last administration, the rats were dissected under ether anesthesia to collect a blood sample and the colon.

### Assessment of diarrhea

Following the single or 7-day repeated dosing schedule, we collected feces that had been excreted from rats within 5 h after the last drug administration to measure the total number of fecal pellets and total fecal weight. The excreted feces were dried for 24 h by vacuum freeze-drying, and the water content per gram of feces was calculated based on the difference between the wet and dry fecal weights.

### Bile acid concentration in blood

Blood collected from rats was centrifuged (1000×*g*, 4 °C, 15 min) to fractionate the plasma. The total bile acid concentration in plasma was then measured using a total bile acid test (Wako Pure Chemical Industries, Ltd., Osaka, Japan).

### Hematoxylin and eosin (HE) staining

Colons isolated from rats were immersed in 10% neutral buffered formalin for fixation. The tissues were embedded in paraffin and sectioned into 3-μm slices that were placed on glass slides. The slides were deparaffinized and stained with hematoxylin followed by eosin. The slides were dehydrated in alcohol, cleared in xylene, and covered for microscopic examination.

### Total RNA preparation and real-time RT-PCR

TRI reagent was added to approximately 20 mg of colon tissue, and total RNA was extracted. A high-capacity cDNA reverse transcription kit was used to synthesize cDNA from 1 μg of RNA.

Real-time PCR was performed using the primers listed in Table 1. The reaction conditions included denaturation at 95 °C for 15 s, annealing at 56 °C for 30 s, and elongation at 72 °C for 30 s. The fluorescence intensity was monitored during the amplification process using the CFX Connect™ Real-Time PCR Detection System (Bio-Rad Laboratories).

**Table 1:** Primer sequences

Gene	Forward (5′–3′)	Reverse (5′–3′)
rTNF-α	GAAACACACGAGACGCTGAAGT	CACTGGATCCCGGAATGTCGAT
rIL-1β	TCAGGCTTCCTTGTGCAAGTGT	ACAGGTCATTCTCCTCACTGTC
rIL-6	TAGTCCTTCCTACCCCAACTTC	GCCGAGTAGACCTCATAGTGAC
rCOX-1	AAGGAGATGGCCGCTGAGTT	AGGAGCCCCCATCTCTATCA
rCOX-2	GCTGATGACTGCCCAACTC	GATCCGGGATGAACTCTCTC
rβ-actin	GCCACTGCCGCATCCTCTTG	CGGAACCGCTCATTGCCGAT

### Extraction of the plasma membrane fraction from the rat colon

The mucosa was scraped from each rat colon sample, suspended in dissecting buffer and homogenized on ice. The homogenate was centrifuged (800×*g* at 4 °C for 15 min), and the resulting supernatant was further centrifuged (17,000×*g* at 4 °C for 30 min). The resulting precipitate included the plasma membrane (PM) fraction with abundant cell membrane [8, 9].

### Western blotting

Each sample was diluted with loading buffer, and after polyacrylamide gel electrophoresis, the proteins were transferred to a polyvinylidene difluoride membrane. After blocking, the membrane was incubated with rabbit anti-rat AQP3 antibody and then with donkey anti-rabbit IgG-HRP. This membrane was reacted with ECL Prime Western blotting detection reagents, and the bands detected by the LAS-3000 mini-imaging system (Fujifilm, Tokyo, Japan) and analyzed.

### Analysis of gut microbiota

DNA was extracted from fecal pellets in the rat colon using the method published by Takahashi et al. [10]. Bacterial 16S rDNA was amplified with the 341f-R806 primer and sequenced using the Illumina MiSeq sequencing system. Based on the determined sequence, we searched RDP MultiClassifier ver. 2.11 (16S rDNA) (confidence: 0.8) for microorganisms identified at the genus level and DB-BA10 (TechnoSuruga Laboratory, Shizuoka, Japan), a microorganism-identifying database, for those identified at the species level with ≥97% homology. The Quantitative Insights Into Microbial Ecology (QIIME) pipeline was used to classify microorganisms with 97% homology in the Greengenes Database (16S rDNA) (confidence 0.5) into an operational taxonomic unit.

### Statistical analysis

Numerical data are presented as the mean ± standard deviation (SD). The significance of differences was examined using Tukey’s test.

## Result

### The laxative effect of repeated oral administration of DKT to rats

**Fig. 2:**
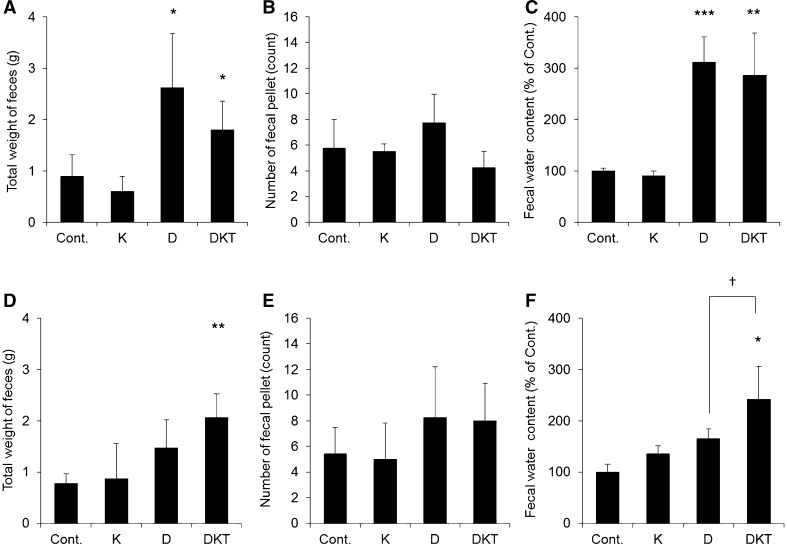
Assessment of diarrhea after Daio, Kanzo, or DKT administration. Kanzo (K), Daio (D) or DKT was administered orally to rats once (**A**–**C**) or for 7 days (**D**–**F**). Total feces weight (**A** and **D**), total number of fecal pellets (**B** and **E**), and fecal water content (**C** and **F**) were measured 5 h after the last treatment. Fecal water content was normalized to the mean value in the control group, which was set to 100% (mean ± SD; *n* = 5; Tukey’s test: *p < 0.05, **p < 0.01, ***p < 0.001 vs Cont.; †*p* < 0.05 vs D)

A single dose of Daio induced diarrhea with increases in total feces weight, total number of fecal pellets, and fecal water content compared to control. However, when Daio was dosed repeatedly for 7 days, the increases in these indexes were all smaller, and the severity of diarrhea was milder than that after a single dose of Daio. These results indicate that Daio causes diarrhea after administration of a single dose but rarely causes diarrhea after repeated administration (Fig. 2). The amount of feeding after Daio treatment was the same as that after control treatment (data not shown).

Interestingly, diarrhea was obvious after a single dose of DKT, although the severity was milder than after a single dose of Daio. Unlike Daio treatment, DKT treatment for 7 days caused a level of diarrhea similar to that caused by a single dose (Fig. 2). The amount of feeding after DKT treatment was the same as after control treatment (data not shown).

We therefore found that repeated administration attenuates the laxative effect of Daio but maintains that of DKT. We also showed that Kanzo itself has no laxative effect.

### Changes in AQP3 expression in the rat colon after repeated administration of DKT

Among the AQP family members in the colon, AQP3 is expressed predominately in mucosal epithelial cells that are in direct contact with the intestinal contents [11, 12]. Previously, we demonstrated that an increase or decrease in AQP3 is involved in the onset of constipation or diarrhea [8, 13] and that oral Daio and its active ingredient sennoside A reduce AQP3 expression to cause a laxative effect in rats [2]. With a focus on AQP3 in the colon, we studied the mechanism underlying the persistent laxative effect of DKT upon repeated administration.

**Fig. 3:**
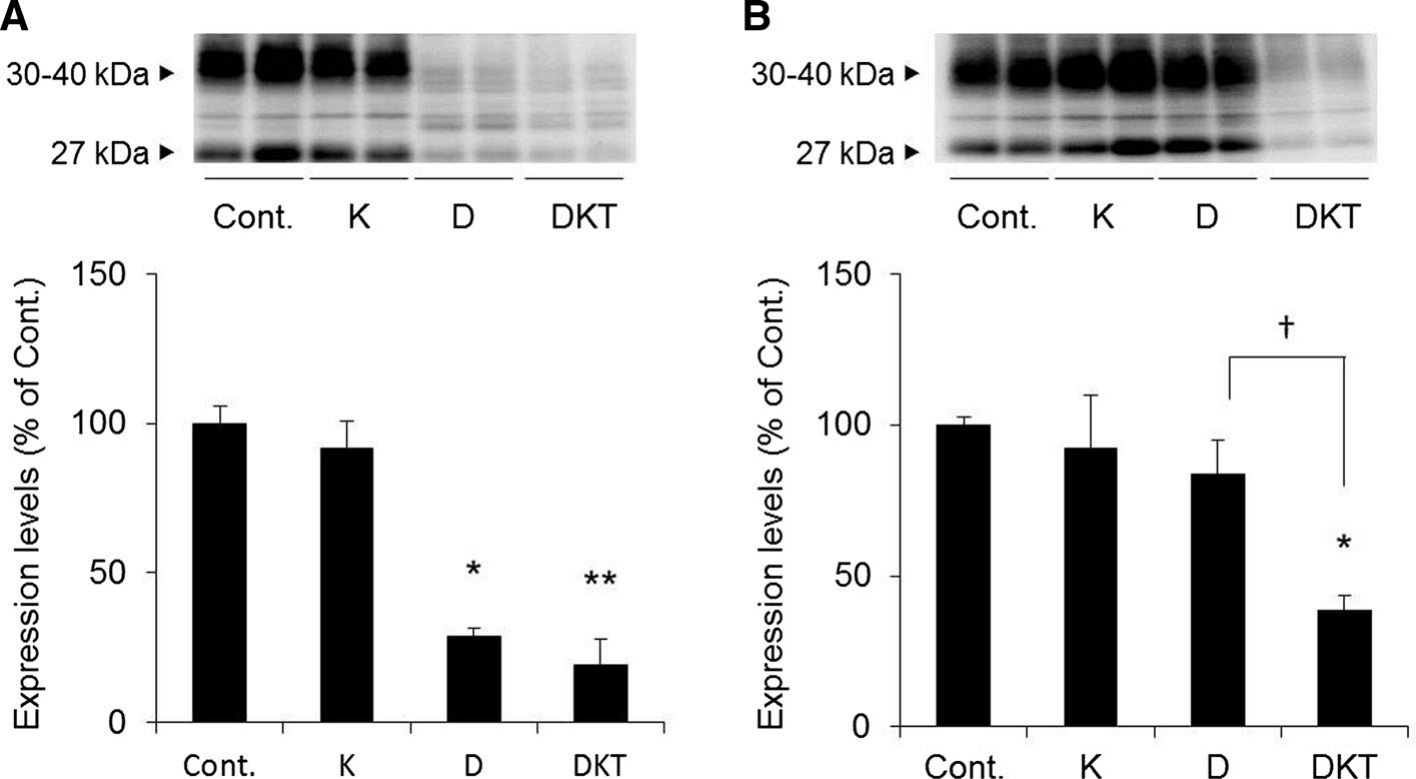
AQP3 protein expression in the rat colon after treatment. Kanzo (K), Daio (D) or DKT was orally administered to rats once (**A**) or for 7 days (**B**), and the colon was collected 5 h after the last treatment. AQP3 protein expression in the PM fraction was detected with Western blotting, and the results were normalized to the mean value in the control group, which was set to 100% (mean ± SD; *n* = 5; Tukey’s test: *p < 0.05, **p < 0.01 vs Cont.; †*p* < 0.05 vs D)

**Fig. 4:**
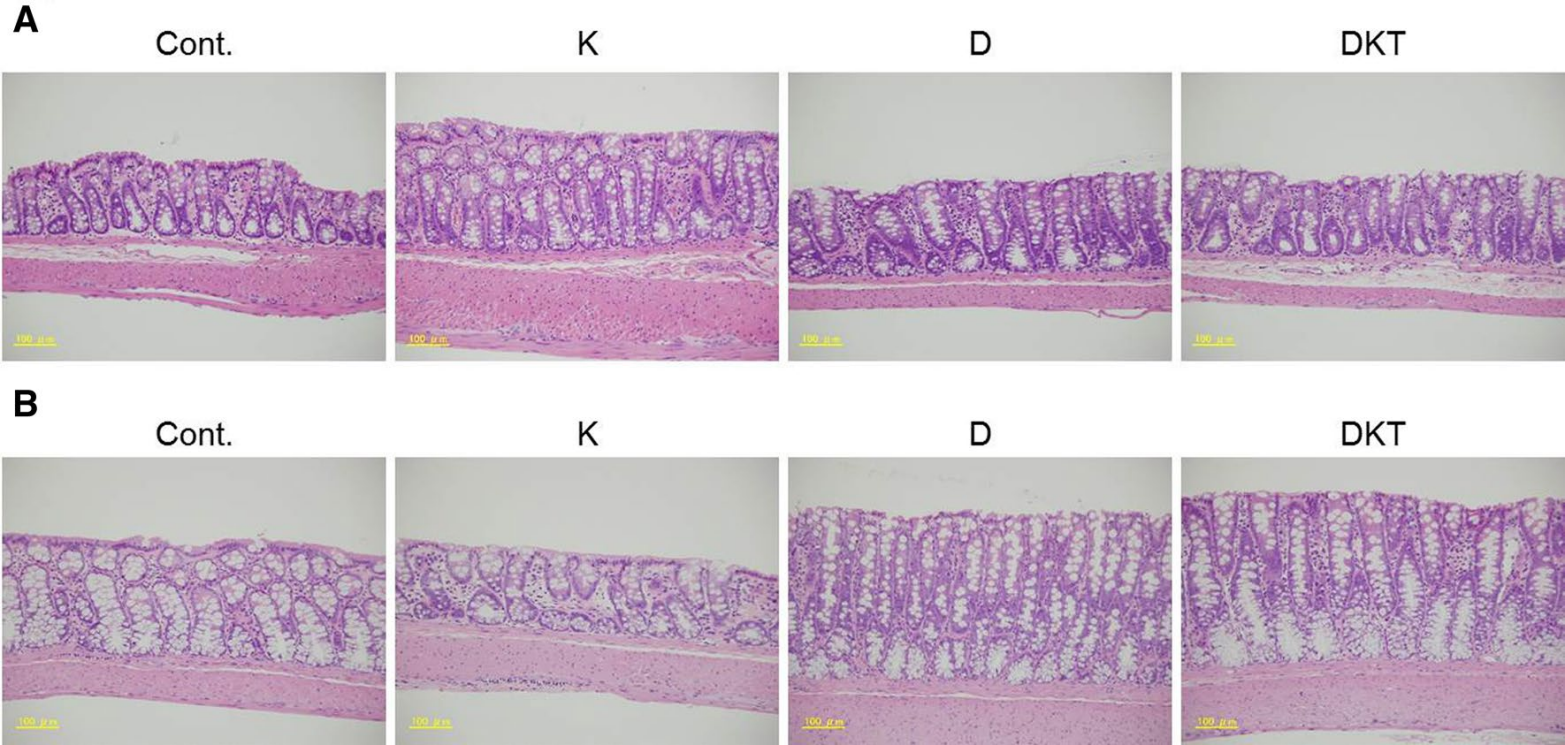
Assessment of tissue morphology after treatment. Kanzo (K), Daio (D), or DKT was administered to rats once (**A**) or for 7 days (**B**), and the colon was collected 5 h after the last treatment. HE staining was performed to examine the condition of the colon

When a single dose of Daio or DKT was administered to rats, AQP3 expression in the colonic membrane fraction was significantly decreased to approximately 30% in both groups compared to the control group (Fig. 3a). With the 7-day repeated dosing schedule, AQP3 expression decreased in the DKT group as it did after a single dose but was not decreased in the Daio (Fig. 3b). AQP3 expression did not change in the Kanzo-treated group. As shown in Fig. 4, there was no histological damage to the colon after repeated administration of Daio, Kanzo or DKT.

These results indicated that decreased AQP3 expression in the colon was maintained by 7-day repeated administration of DKT but not of Daio. We also noted that Kanzo itself does not reduce AQP3 expression. In addition, histological damage to the colon is not involved in the observed changes.

### Changes in the inflammatory response in the colon after DKT treatment

We have shown that decreased AQP3 expression in the colon after sennoside A treatment is related to inflammation mediated by activated colonic macrophages [2]. Repeated administration of lipopolysaccharide (LPS) to mice was reported to gradually attenuate the LPS-induced inflammatory response [14–16]. We hypothesized that DKT, which contains the anti-inflammatory agent Kanzo, could control the inflammation caused by Daio, thereby maintaining the laxative effect and the decreased AQP3 expression. Therefore, we investigated the changes in the inflammatory response in the rat colon after a single dose or a 7-day course of Daio or DKT.

**Fig. 5:**
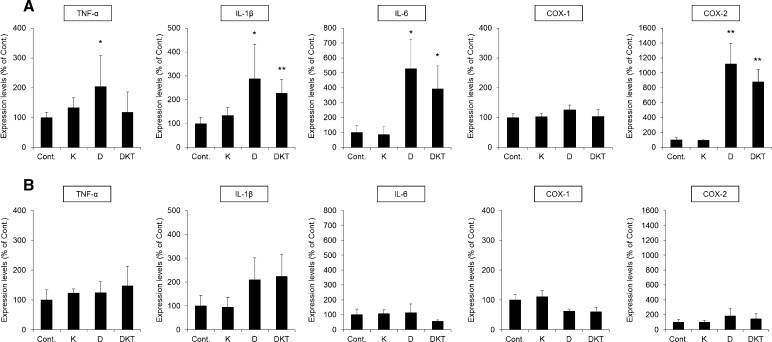
The mRNA expression of inflammatory cytokines and COX enzymes in the rat colon after treatment. Kanzo (K), Daio (D), or DKT was administered orally to rats once (**A**) or for 7 days (**B**), and the colon was collected 5 h after the last treatment. The mRNA expression of TNF-α, IL-1β, IL-6, COX-1, and COX-2 was analyzed using real-time PCR. The results were normalized to those of β-actin, which were indexed by setting the mean value in the control group to 100% (mean ± SD; *n* = 5; Tukey’s test: *p < 0.05, **p < 0.01 vs Cont.)

In rats given a single dose of DKT, the mRNA expression of interleukin (IL)-1β and IL-6 in the colon increased significantly compared to that in control rats. However, there was no change in the expression of COX-1, a resident enzyme, but the expression of COX-2, an enzyme induced by inflammation, was significantly increased. After Daio treatment, the mRNA expression of inflammatory cytokines and COX-2 was significantly increased (Fig. 5a).

After 7 days of repeated administration of Daio or DKT, the mRNA expression of tumor necrosis factor-α (TNF-α), IL-1β, IL-6 and COX-2 in the colon was not significantly different from that after control treatment (Fig. 5b).

These results suggest that an inflammatory response is not involved in the persistent laxative effect or the decreased AQP3 expression in the colon observed with repeated DKT administration. These findings also indicate that the attenuated laxative effect observed after repeated Daio administration is less likely to be caused by reduced macrophage-mediated inflammation.

### Changes in total bile acid in blood after repeated DKT administration

Previous results indicate that repeated DKT administration causes a laxative effect by decreasing AQP3 expression in the colon and that this effect is not caused by inflammation. Increased bile acid in blood or feces, or a change in its composition, plays a role in the onset of diarrhea [17, 18]. Yde et al. [19] demonstrated the onset of diarrhea in rats given bile acid, with decreased AQP3 expression in the colon. Furthermore, it has been reported that the total bile acid concentration in blood is increased when bile acid-induced diarrhea occurs [20]. Based on these findings, we hypothesized that repeated DKT administration changes the amount or composition of bile acid, leading to decreased AQP3 expression in the colon. Therefore, we measured the total bile acid concentration in blood after repeated DKT administration.

**Fig. 6:**
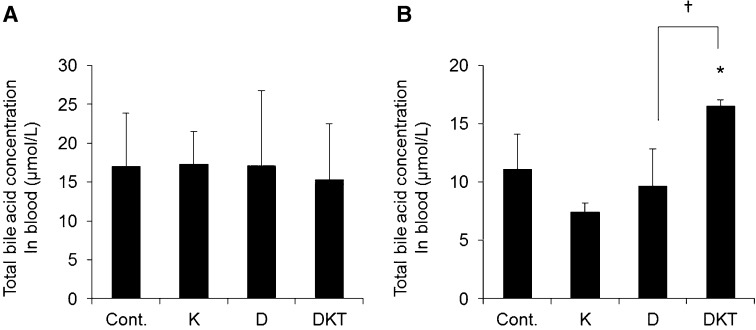
Total bile acid concentration in blood after treatment. The total bile acid concentration in blood was measured in rats given Kanzo (K), Daio (D), or DKT once (**A**) or for 7 days (**B**) (mean ± SD; *n* = 5; Tukey’s test: *p < 0.05 vs Cont.; †*p* < 0.05 vs D)

The total bile acid concentration in blood from rats given a single dose of Daio or DKT was not significantly different from the control rats (Fig. 6a). When DKT was administered to rats repeatedly for 7 days, the total bile acid concentration in blood increased significantly compared to the control rats, but there were no changes with repeated administration of Daio or Kanzo (Fig. 6b).

These results suggest that the total bile acid concentration in blood does not change after repeated administration of Daio for 7 days but does increase with repeated administration of DKT.

### Changes in gut microbiota after repeated administration of DKT

The amount and composition of bile acid is affected by changes in enteric bacteria, including *Lactobacillus*, *Bacteroides*, and *Bifidobacterium* [21–25]. It has been reported that Daio has antibacterial activity against *Bacteroides* [26] and that glycyrrhizin and liquiritin in Kanzo changed the amount of *Clostridium* and *Enterococcus* [27, 28]. We therefore analyzed the gut microbiota after repeated administration of DKT using next-generation sequencing.

**Fig. 7:**
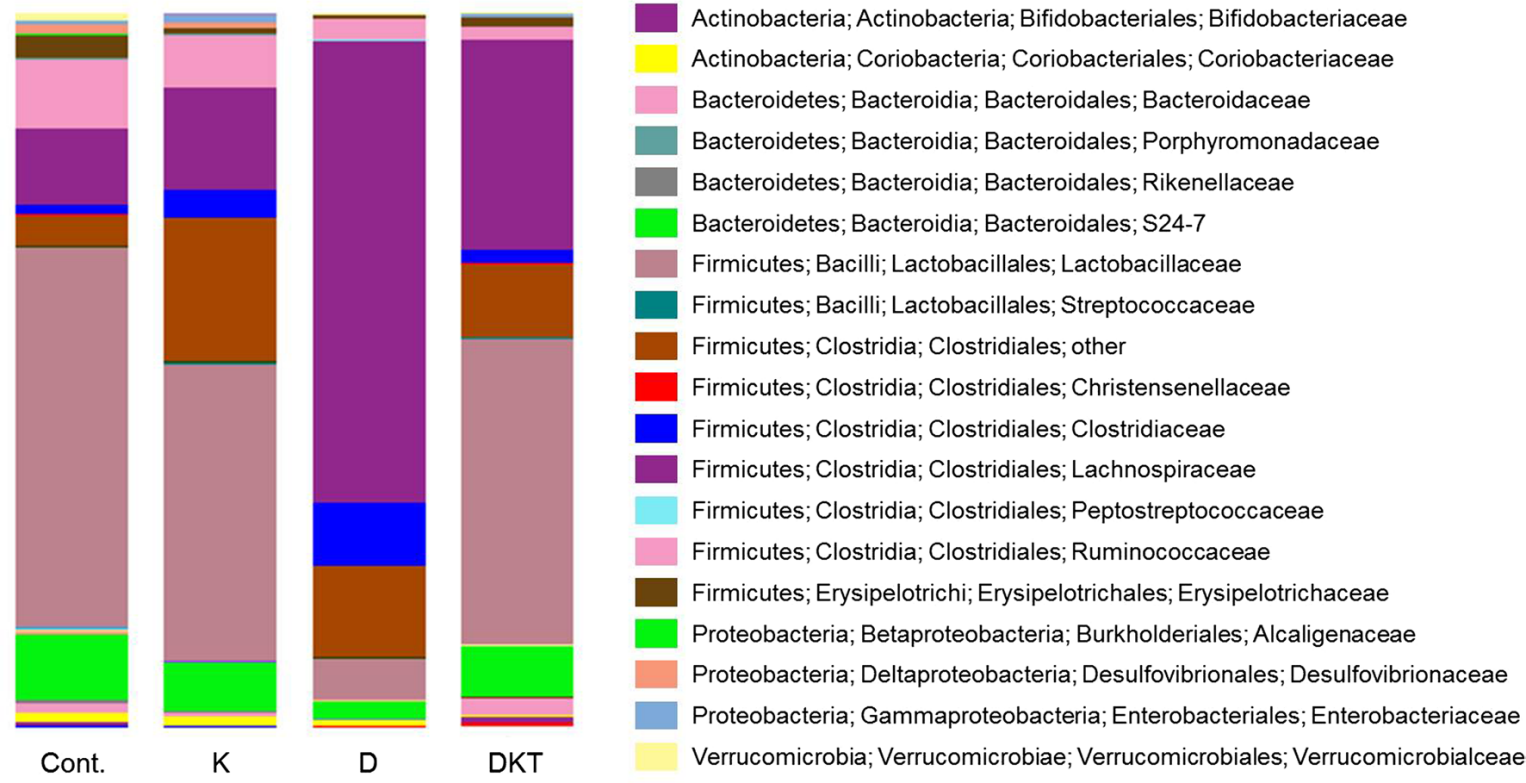
Relative abundance of gut microbiota after treatment for 7 days. The relative abundance of gut microbiota in feces of rats given Daio (D), Kanzo (K), or DKT for 7 consecutive days was analyzed by next-generation sequencing

After repeated administration of DKT for 7 days, *Lactobacillaceae* accounted for approximately 50% of the gut microbiota, followed by *Lachnospiraceae*. These bacteria made up approximately 70% of the total bacteria population, and these results were similar to those in the control group. After repeated administration of Daio, the abundance of *Lachnospiraceae* was approximately 65%, and that of *Lactobacillaceae* decreased significantly, suggesting a greater change in the microbiota than after DKT treatment (Fig. 7 and Table 2).

**Table 2:** Family level component ratio (%) of microbiota after treatment for 7 days

Family	Cont.	Kanzo	Daio	DKT
*Bifidobacteriaceae*	0.5	0.1	0.1	0.6
*Coriobacteriaceae*	1.4	1.4	0.7	0.2
*Bacteroidaceae*	1.3	0.4	0.1	2.3
*Porphyromonadaceae*	0.1	0.1	0	0.2
*Rikenellaceae*	0.5	0.2	0	0
*S24*-*7*	9.1	6.7	2.5	7.1
*Lactobacillaceae*	53.1	41.6	5.8	42.9
*Streptococcaceae*	0.1	0.2	0	0.1
*Christensenellaceae*	0.2	0.1	0.1	0
*Clostridiaceae*	1.3	3.8	8.7	1.9
*Lachnospiraceae*	10.6	14.1	64.5	29.4
*Peptostreptococcaceae*	0	0	0.3	0
*Ruminococcaceae*	9.6	7.5	2.8	1.9
*Clostridiales (o) other*	4.5	20.3	12.9	10.3
*Erysipelotrichaceae*	3	0.7	0.4	1.3
*Alcaligenaceae*	0.3	0.1	0.1	0.1
*Desulfovibrionaceae*	1.3	0.6	0	0
*Enterobacteriaceae*	0.4	1.3	0.1	0.4
*Verrucomicrobiaceae*	1	0.1	0.2	0.2

The abundance of *Bacteroidaceae* increased approximately twofold after 7 days of DKT treatment but decreased to approximately one-tenth after repeated administration of Daio compared to control treatment. For *Bifidobacteriaceae*, the abundance was similar in the DKT-treated group and the control group but was decreased in the Daio-treated group (Fig. 6 and Table 2).

These results show that the abundance of major bacteria changed considerably after repeated Daio administration but remained similar to that in the control group after repeated DKT administration.

## Discussion

In this study, we investigated the laxative effects of repeated administration of DKT and of Daio to establish scientific evidence for DKT as a therapeutic for chronic constipation and clarified the role of Kanzo in DKT.

In rats given a single dose of DKT, fecal water content was significantly increased compared to that in the control group, and AQP3 expression in colonic mucosal epithelial cells was decreased. Similar changes were observed after a single dose of Daio (Figs. 2, 3). We therefore concluded that the lack of a difference in the laxative effects of a single dose of DKT and Daio stemmed from the similar decrease in AQP3 expression in the colon after treatment. When DKT was administered to rats repeatedly for 7 days, diarrhea and decreased AQP3 expression in the colon were observed. When Daio was administered repeatedly, there was not a significant increase in fecal water content and AQP3 did not change compared to the control group. Kanzo itself had no laxative effect and did not decrease the colonic expression of AQP3. These results suggest that DKT, unlike Daio alone, can maintain its laxative effect upon repeated dosing, likely due to a persistent decrease in AQP3 expression in the colon.

We then investigated the mechanism underlying the persistent decrease in AQP3 expression upon repeated administration of DKT. AQP3 expression decreases in response to inflammation [29, 30]. A single dose of Daio or DKT probably evoked a laxative effect through the inflammation-mediated decrease in AQP3. After a 7-day treatment with Daio or DKT, we did not observe any inflammation or mucous disorders (Figs. 4, 5). It is therefore less likely that the decreased AQP3 expression observed after continued use of DKT is related to the inflammatory response.

Diarrhea occurs when excess bile acid flows into the large intestine due to its malabsorption or changes in its composition/synthesis; this phenomenon is called bile acid-induced diarrhea [17]. It was recently reported that AQP3 expression in the colon is decreased in bile acid-induced diarrhea [19]. We therefore considered that the development of diarrhea and decreased AQP3 expression in the colon upon repeated administration of DKT were probably caused by bile acid. In fact, we found that the total bile acid concentration in blood was increased only upon repeated dosing of DKT (Fig. 6). In addition, the abundance of *Bacteroidaceae*, which is involved in the deconjugation of conjugated bile acids, was increased after repeated administration of DKT (Fig. 7 and Table 2). Taurocholic acid (TCA), a conjugated bile acid, negatively controls bile acid synthesis [31]. In the presence of DKT, *Bacteroidaceae* probably cause a decrease in conjugated bile acids (e.g., TCA), and bile acid synthesis increases in response, resulting in decreased expression of AQP3. Although the mechanism whereby bile acid decreases AQP3 expression is still unknown, it may involve the direct action of bile acids or factors whose production is influenced by bile acids, such as glucagon-like peptide-1 or enterobacteria-derived fatty acids.

*Lachnospiraceae* were markedly increased and *Lactobacillaceae* were significantly decreased after repeated administration of Daio, but such changes were not observed after treatment with DKT, probably because Kanzo in DKT inhibits the effects of Daio to increase *Lachnospiraceae* and decrease *Lactobacillaceae*. *Lachnospiraceae* are classified into *Clostridium* cluster XIVa, which is a bacterial group that produces butylate [32–34], which increases AQP3 (Supplementary Fig. 1). The failure to maintain the decreased AQP3 expression in the colon with continued administration of Daio might be related to increased butylate production by *Lachnospiraceae*.

In summary, DKT, which contains Kanzo, was able to maintain its laxative effect even when used repeatedly. We concluded that the laxative effect is due to the persistent decrease in AQP3 expression in the colon. Gut microbiota homeostasis may be involved in regulating AQP3 expression. Our study results indicate that DKT can be used for a long period to treat chronic constipation. This study also provides new evidence regarding the significance of Kampo compounds.

## Electronic supplementary material

Below is the link to the electronic supplementary material.

**Supplementary Fig.** **1.** Effect of butylate on AQP3 mRNA expression in HT-29 cells. HT-29 cells were treated with butylate (0–400 μM), and AQP3 mRNA expression in cells cultured for 6 h was analyzed using real-time PCR. The results were normalized to those of GAPDH, which were indexed by setting the mean value in the control group to 100% (mean ± SD; *n* = 5; Dunnett’s test: **p < 0.01, ***p < 0.001 vs 0 μM) (TIFF 9616 kb)
