# Supplementary figures and images for: Laxative effect of repeated Daiokanzoto is attributable to decrease in aquaporin-3 expression in the colon
Source: J Nat Med. 2018 Jan 27;72(2):493–502. doi: 10.1007/s11418-018-1174-1 (PMC6469848; doi:10.1007/s11418-018-1174-1)

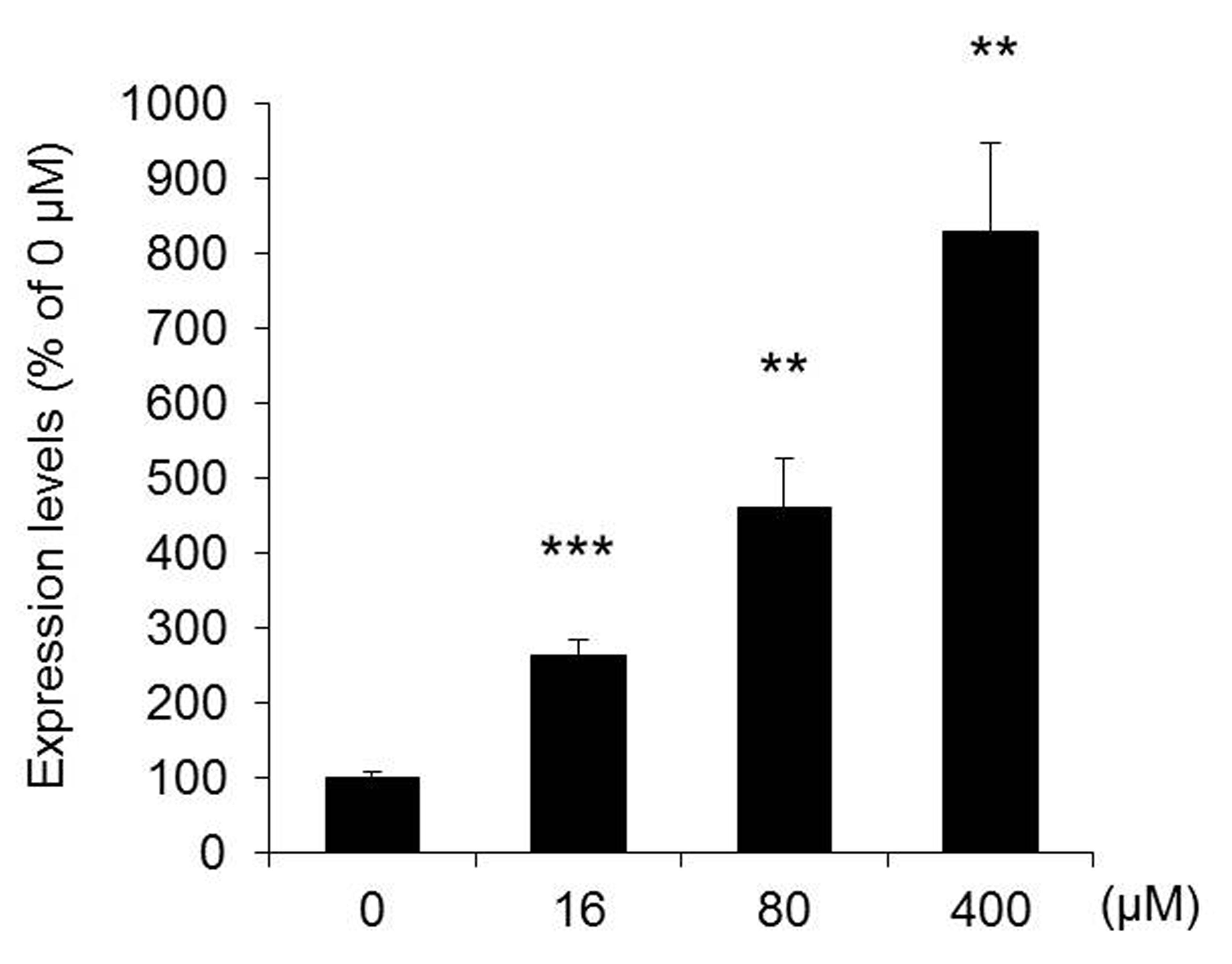

Supplement: Supplementary file 1 — Supplementary Fig. 1. Effect of butylate on AQP3 mRNA expression in HT-29 cells. HT-29 cells were treated with butylate (0–400 μM), and AQP3 mRNA expression in cells cultured for 6 h was analyzed using real-time PCR. The results were normalized to those of GAPDH, which were indexed by setting the mean value in the control group to 100% (mean ± SD; n = 5; Dunnett’s test: **p < 0.01, ***p < 0.001 vs 0 μM) (TIFF 9616 kb) [file 11418_2018_1174_MOESM1_ESM.tif]
